# Fitness for purpose of routinely recorded health data to identify patients with complex diseases: The case of Sjögren's syndrome

**DOI:** 10.1002/lrh2.10242

**Published:** 2020-09-08

**Authors:** Sytske Wiegersma, Linda E. Flinterman, Chiara Seghieri, Chiara Baldini, John Paget, Jaime Barrio Cortés, Robert A. Verheij

**Affiliations:** ^1^ Netherlands Institute for Health Services Research (NIVEL) Utrecht The Netherlands; ^2^ Institute of Management Sant'Anna School of Advanced Studies Pisa Italy; ^3^ Department of Clinical and Experimental Medicine University of Pisa Pisa Italy; ^4^ Faculty of Education and Health University Camilo José Cela Madrid Spain; ^5^ Tilburg School of Social and Behavioral Sciences Tilburg University Tilburg The Netherlands

**Keywords:** data linkage, electronic health record, patient selection algorithm, primary care, primary Sjögren's syndrome, secondary care

## Abstract

**Background:**

This study is part of the EU‐funded project HarmonicSS, aimed at improving the treatment and diagnosis of primary Sjögren's syndrome (pSS). pSS is an underdiagnosed, long‐term autoimmune disease that affects particularly salivary and lachrymal glands.

**Objectives:**

We assessed the usability of routinely recorded primary care and hospital claims data for the identification and validation of patients with complex diseases such as pSS.

**Methods:**

pSS patients were identified in primary care by translating the formal inclusion and exclusion criteria for pSS into a patient selection algorithm using data from Nivel Primary Care Database (PCD), covering 10% of the Dutch population between 2006 and 2017. As part of a validation exercise, the pSS patients found by the algorithm were compared to Diagnosis Related Groups (DRG) recorded in the national hospital insurance claims database (DIS) between 2013 and 2017.

**Results:**

International Classification of Primary Care (ICPC) coded general practitioner (GP) contacts combined with the mention of “Sjögren” in the disease episode titles, were found to best translate the formal classification criteria to a selection algorithm for pSS. A total of 1462 possible pSS patients were identified in primary care (mean prevalence 0.7‰, against 0.61‰ reported globally). The DIS contained 208 545 patients with a Sjögren related DRG or ICD10 code (prevalence 2017: 2.73‰). A total of 2 577 577 patients from Nivel PCD were linked to the DIS database. A total of 716 of the linked pSS patients (55.3%) were confirmed based on the DIS.

**Conclusion:**

Our study finds that GP electronic health records (EHRs) lack the granular information needed to apply the formal diagnostic criteria for pSS. The developed algorithm resulted in a patient selection that approximates the expected prevalence and characteristics, although only slightly over half of the patients were confirmed using the DIS. Without more detailed diagnostic information, the fitness for purpose of routine EHR data for patient identification and validation could not be determined.

## 1 INTRODUCTION

### 1.1 The diagnosis of Sjögren's syndrome

Primary Sjögren's syndrome (pSS) is an underdiagnosed, long‐term autoimmune disease that affects particularly salivary and lachrymal glands but that may involve any organ and system.^1^ Despite generally benign, pSS may be characterized by severe rare complications including non‐Hodgkin's lymphoma (NHL) with an unneglectable impact on patients' quality of life.^2^, ^3^ To date, health policy and management research for pSS are quite rare, especially on pSS diagnosis and management in primary health care.^4^

A study on the epidemiology of Sjögren's syndrome by Patel and Shahane^5^ concluded that: “there is no accepted universal classification criterion for the diagnosis of Sjögren's syndrome. There are a limited number of studies that have been published on the epidemiology of Sjögren's syndrome, and the incidence and prevalence of the disease varies according to the classification criteria used. The data is further confounded by selection bias and misclassification bias, making it difficult for interpretation.” [p. 247]. In fact, international consensus on the classification criteria for pSS was only reached in 2016, resulting in the American College of Rheumatology/European League Against Rheumatism (ACR/EULAR) classification criteria for pSS,^6^ making it difficult to estimate the exact prevalence of the disease. Consequently, estimates of the prevalence of pSS vary greatly across studies (ranging from 0.11‰ to 37.9‰), depending on the setting and the definition used and the population investigated.^7^

Besides population, geographical, and diagnostic differences, diagnosis may be delayed or patients may be misclassified as another rheumatic disease due to the insidious onset and the broad spectrum of clinical manifestations of the disease. In addition, Sjögren's Syndrome (SS) can occur on its own (primary SS) or in association with other systemic autoimmune diseases (secondary SS). Given the vast availability of electronic health records (EHRs) for the general population, computational phenotyping may help to improve the diagnosis and timely referral of patients with complex diseases such as pSS to the medical specialist. Computational phenotyping algorithms are automated patient selection algorithms to identify a patient population of interest.^8^ Such algorithms are increasingly used to identify and characterize patients with complex medical conditions from heterogeneous EHR data in order to improve efficiency of healthcare delivery and clinical outcomes.^9^

### 1.2 Primary care data

Primary care EHRs are a rich source of information about people's health and health service utilization. In countries with a gatekeeping system, general practitioners (GPs) have a fixed practice population and they are normally the first point of contact with the health care system. Routinely recorded electronic health care data in primary care may be used to develop early detection models or estimate population prevalences for diseases such as pSS defined as “complex with rare complications”^10^ and in general, to study the disease in a “real life” situation, outside the setting of a specialized clinical center.^11^

In the Netherlands, and in many other countries in Europe (eg, the United Kingdom, Italy, and Spain), primary care practices use an EHR system to record the care delivered to their patients and the health problems presented.^12^ The diagnoses that are recorded can be assessed by the GP, but also in other sectors of the health care system, such as medical specialists. For many diseases, GPs are unable to diagnose the patients themselves so patients are referred to a medical care specialist for diagnosis and treatment. Diagnoses recorded in the GP EHR data are therefore not necessarily diagnoses made by the GPs but also include those of other healthcare specialists.

Two characteristics make it worthwhile to investigate primary care EHR data in relation to Sjögren's syndrome:

1. The GP is the first point of contact with the health care system. This allows us to identify the patient's first symptoms and to analyze the care trajectories that eventually lead to the diagnosis of Sjögren and its treatment in primary care and eventually in secondary care.
2. There is a fixed patient list. This means that the data recorded in primary care are population based and that there is an epidemiological denominator available.

One of the difficulties in identifying patients with pSS, or any other relatively rare disease, from EHRs is the coding system used in primary care. GPs in the Netherlands use the International Classification of Primary Care (ICPC) coding system to record diagnoses and symptoms. The ICPC coding system was especially devised for primary care settings.^13^ In contrast with for example the International Classification of Diseases (ICD^14^) coding system used in secondary care, ICPC has separate entries for symptoms (such as belly ache) and for diagnoses (such as urinary tract infection). However, as there are only about 700 separate entries, the level of granularity of ICPC coded primary care records is lower than that of the ICD coded records in secondary care.^15^

Due to the low granularity of the ICPC coding system, there is no separate ICPC code for pSS. pSS is recorded under “Musculoskeletal disease other (L99),” as are for example Systemic Lupus Erythematosus and Systemic Sclerosis, which are autoimmune disorders that can occur in association with Sjögren's syndrome.^16^ An important consequence for our purposes is the fact that there is no simple way to identify pSS patients from primary care EHRs and no gold standard available to validate any patient selection made based on alternative rules or criteria. However, this information may be available from other sources, such as insurance claims data from secondary care.

### 1.3 Secondary care data

As the GP is the first point of contact in the Netherlands, undiagnosed patients will first visit their GP with any complaints typical for Sjögren's syndrome. When the GP suspects Sjögren's syndrome, the GP will refer the patient to the Rheumatologist, Internist, or Ophthalmologist for specialized care and diagnosis. After formal diagnosis, general care for Sjögren's patients consists of follow‐up appointments (medical checkups) with the medical specialist and symptomatic treatment (eg, artificial tears or artificial saliva to reduce the symptoms of drought). After first prescription of these drugs by the specialist, repeat prescriptions are generally prescribed by the GP. The medical specialist informs the GP of the diagnosis made, which is then included by the GP in the patient's primary care EHR. The fact that all suspected Sjögren's patients are eventually referred to secondary care for diagnosis and treatment means that all Sjögren's patients should ultimately show up in secondary care records. Diagnostic information can be retrieved from hospital claims data using two classification systems; the diagnosis related groups (DRG) for hospital reimbursements and aforementioned ICD coding system for diseases. Both systems contain explicit codes for Sjögren's disease.

This study investigates to what extent routinely recorded EHR data can be used to identify patients with complex diseases. To this aim we first examined how formal inclusion and exclusion criteria for pSS could be translated into a computational phenotyping algorithm to identify pSS patients in primary care. As the primary care data do not contain a gold standard to validate the algorithm, we secondly assessed whether secondary care data could be used as an alternative validation method, by comparing the resulting patient selection with DRG and ICD codes retrieved from hospital claims data. In order to assess the overall fitness for purpose of routinely recorded health care data for the identification of patients with complex diseases such as pSS, we finally compared prevalence rates and patients' demographic characteristics to those reported in literature.

## 2 METHODS

### 2.1 Data sets

#### 2.1.1 General practitioner electronic health records

Nivel is a research institute that is part of the Dutch national health knowledge infrastructure. Nivel is commissioned by the Dutch Ministry of Health to collect data from EHRs in primary care, in Nivel Primary Care Database (Nivel PCD). Nivel PCD collects routinely recorded data from health care providers to monitor the health of patients and the utilization of health services in a representative sample of the Dutch population. Data are extracted periodically, and patients can be followed through the health care system longitudinally when the Nivel data are linked to other national databases.

For this study, data were extracted for the years 2006 to 2017, containing consultations, diagnoses, prescriptions, referrals, and patient characteristics.^17^ Diagnoses are recorded routinely in general practices and GPs use the ICPC classification system. Due to privacy regulations, the database contains no information stored in free text fields, apart from the titles of the disease episodes. This project has been approved by the governance bodies of Nivel PCD under No. NZR‐00317.057.

#### 2.1.2 Hospital claims database

The national claims data set is provided by Diagnosis Related Groups Information System (DIS) and is accessible and linkable through Statistics Netherlands, a government institution that makes data available for policy development and scientific research. The data set includes claims data, using the DRG classification system for hospital reimbursements,^18^ for all hospitals in the Netherlands.

DRG codes were available for the years 2013 to 2017 at the time of research (November 2019). DRG codes for Sjögren's syndrome are recorded under three medical specialisms; Rheumatology (DRG code 0324‐03‐00‐0308), Internal Medicine (DRG code 0313‐05‐00‐0524), and Ophthalmology (DRG code 0301‐40‐00‐0404). For the most recent years (2016‐2017), ICD‐10 codes are increasingly available, although not complete. The ICD‐10 code for Sjögren's syndrome is M35.0 (sometimes recorded as M350).

#### 2.1.3 Population

In the Netherlands, all non‐institutionalized inhabitants are compulsorily listed with a general practice, even if they do not visit their GP regularly. Nivel PCD contains primary care data of 1.7 million individuals (10% of the Dutch population), enlisted in approximately 500 GP practices. The practices included in Nivel PCD and patients enlisted in each practice may vary over the years. Patients can be tracked over time and linked to other sources based on pseudonymized citizen numbers. In total we analyzed the EHRs of 3 056 928 unique patients enlisted in any practice included in Nivel PCD over the years 2006 to 2017. The DIS database contains DRG‐coded insurance claims data for 12 991 265 unique patients who consulted a medical specialist in the Netherlands between 2013 and 2017.

### 2.2 Developing the algorithm

The first aim of this study was to assess whether primary care electronic health care data could be used to identify pSS patients from primary care electronic health care records. The formal ACR/EULAR classification criteria for pSS were used as a starting point to define inclusion and exclusion criteria for patient selection, but additional information available from the primary care database, such as drug prescriptions and disease episode titles, was also explored.

#### 2.2.1 Formal classification criteria for Sjögren's syndrome

##### Inclusion criteria

The ACR/EULAR criteria^6^ include patients who report at least one symptom of ocular or oral dryness and score above a certain threshold on certain weighted criteria items. Ocular or oral dryness is assessed by diagnostic questions regarding recent eye complaints, use of artificial tears, reporting of dry mouth, and difficulty swallowing food. The weighted criteria concern labial salivary gland histopathology, anti‐SSA/Ro antibodies, ocular staining score, Schirmer's test, and unstimulated whole saliva flow rate.

##### Exclusion criteria

The ACR/EULAR criteria^6^ exclude patients with a prior diagnosis of the conditions: history of head and neck radiation treatment, Active hepatitis C infection (with confirmation by polymerase chain reaction), AIDS, Sarcoidosis, Amyloidosis, Graft‐vs‐host disease, or IgG4‐related disease.

##### Secondary Sjögren's syndrome

In order to distinguish specifically primary Sjögren's syndrome, Systemic Lupus Erythematosus, Systemic Sclerosis, and Rheumatoid Arthritis should additionally be excluded.^16^

#### 2.2.2 Data recorded in primary care

To identify the possible pSS patients, the formal criteria were translated into a set of rules relating to coded diagnoses, comorbidities, and diagnostic test results. We additionally explored drug prescriptions and disease episode titles. These rules were applied in the form of automated queries on the database. Except for the disease episodes title, no free text fields could be used.

##### ICPC codes

In secondary care the patients with pre‐specified diseases can be included and excluded using ICD‐10 codes. To apply the ACR/EULAR criteria to primary care data, the ICD‐10 codes were converted to the corresponding ICPC codes using the WHOFIC Thesaurus ICPC2‐ICD10.^19^ The resulting ICPC‐codes were applied to ICPC‐coded GP contacts (eg, consults, prescriptions) and disease episodes.

##### Diagnostic test results

The ACR/EULAR criteria^6^ mention several diagnostic tests that can aid in the diagnosis of pSS. Although Nivel PCD contains a range of diagnostic test results, these cover only the results of tests issued or conducted by GPs. Diagnostic test results are recorded in Nivel PCD using NHG lab codes, defined by the Nederlands Huisartsen Genootschap (Dutch College of General Practitioners) for the classification of laboratory and other diagnostic tests and results.^20^ It was checked how many of the diagnostic tests defined by Shiboski et al^6^ were recorded in Nivel PCD.

##### Prescriptions

The prescriptions in Dutch primary care are coded using the international Anatomical Therapeutic Chemical (ATC) Classification system for medicines.^21^ In order to strengthen the patient selection, we examined the use of certain medication known to be much used by pSS patients.^22^

These are:

- Artificial tears (ATC S01XA20)
- Hydroxyclorochine (ATC P01BA02)
- Cortisone (ATC H02AB10/S01BA03)
- Pilocarpine (ATC N07AX01)
- Ciclosporin (ATC S01XA18)

Especially the combined use of Artificial tears, Hydroxychlorochine, and Pilocarpine was expected to be a strong indicator of pSS.

##### Disease episode titles

Finally, a text query was applied to all disease episode titles recorded between 2006 and 2017. The text query was based on a number of variations in spelling of the word “Sjögren” (namely “sjogren,” “sjorgen,” “sjogern,” “sjögren,” “sjorgren,” “sjoegren,” “sogren”) in the disease episode titles in Nivel PCD. The text strings of found cases were then manually checked and scored as to whether they described primary Sjögren syndrome by two of the authors as: (a) “primary Sjögren”; (b) “perhaps primary Sjögren”; or (c) “not Sjögren” or explicitly “secondary Sjögren.” All cases in which the term Sjögren was followed by a question mark were assigned to category 2. Cases explicitly described as secondary were scored as category 3. This, however, does not necessarily mean that all cases with score 1 are indeed primary Sjögren cases.

### 2.3 Validating the algorithm

As there is no formal diagnosis available to use as a gold standard to validate the developed algorithm, the second aim of this study was to assess to what extent hospital claims data, which contain more fine‐grained DRG treatment and ICD‐10 diagnosis codes for Sjögren, might be suitable as an alternative validation method. We additionally compared prevalence rates and demographic characteristics of pSS patients identified in primary and secondary care with those reported in literature.

#### 2.3.1 Data linkage

EHR data of patients from Nivel PCD were linked to insurance claims data available from the DIS database on the basis of pseudonymized national citizen numbers. A Sjögren related DRG or ICD‐10 code was regarded as a formal diagnosis used to confirm whether pSS patients in found in primary care were also recorded as pSS patients in secondary care.

Because Nivel PCD covers 10% of the Dutch population and the DIS database covers 100% of the Dutch population, it was expected that 10% of the patients found in the DIS database would be retrieved from Nivel PCD. Linkage is done using the patients' citizen service number (BSN), a unique personal number allocated to every registered Dutch citizen. The BSN is used by all recognized care providers, such as GPs, hospitals, and health insurance companies, to identify patients that need care. The BSN is included in Nivel PCD since the year 2014 and as such is not known for patients who did not consult the GP after 2013. For these patients, linkage on BSN level is not possible, leading to a linkage loss of around 10%.

#### 2.3.2 Validation scores

Based on the linked data set, it is possible to compare the pSS patients found with the algorithm from Nivel PCD with formal diagnoses based on recorded DRGs and ICD‐10 codes related to Sjögren within the health insurance claims data set. Based on the combined data sets each patient is flagged as a true positive, true negative, false positive, or false negative, as shown in Table 1:

- True positives (Tp): labeled as pSS by the algorithm, confirmed based on DRG codes secondary care claims database.
- True negatives (Tn): not labeled as pSS by the algorithm (hence not included in our data set), confirmed based on absence of DRG code related to Sjögren recorded in the secondary care claims data.
- False positives (Fp): labeled as pSS by the algorithm but not confirmed based on DRG codes secondary claims database.
- False negatives (Fn): not labeled as pSS by the algorithm, but DRG codes related to Sjögren recorded in the secondary claims database.

**TABLE 1:** Comparison of identified patients in Nivel and DIS databases

Patient	pSS Nivel PCD	pSS DIS database	Check
1	Yes	No	Fp
2	Yes	Yes	Tp
…	No	Yes	Fn
X	No	No	Tn

Abbreviations: DIS, Dutch National Insurance Claims Database; Fn, false negatives; Fp, false positives; Nivel PCD, Nivel Primary Care Database; pSS, primary Sjögren's syndrome; Tn, true negatives; Tp, true positives.

The total number of Tps, Tns, Fps, and Fns can be used to calculate the accuracy and other performance scores of the algorithm:

- $\text{Accuracy}=\frac{\mathrm{Tp}+\mathrm{Tn}}{\text{Total}}$
- $\text{Sensitivity}\;\left(\text{recall}\right)=\frac{\mathrm{Tp}}{\mathrm{Tp}+\mathrm{Fn}}$
- $\text{Specificity}=\frac{\mathrm{Tn}}{\mathrm{Tn}+\mathrm{Fp}}$
- $\text{Postive Pedictive Value}\;\left(\mathrm{PPV},\text{precision}\right)=\frac{\mathrm{TP}}{\mathrm{Tp}+\mathrm{Fp}}$
- $N\text{egative Predictive Value}\;\left(\mathrm{NPV}\right)=\frac{\mathrm{Tn}}{\mathrm{Fn}+\mathrm{Tn}}$
- *F*_1_‐score $=2\times \frac{\text{precision}\times \text{recall}}{\text{precision}+\text{recall}}$

#### 2.3.3 Prevalence rates

The prevalence rates are reported from the year 2011. The current Nivel PCD started in 2010, but as this was still a transition year, data for 2010 should be used with caution. The former database (known as the LIN [Netherlands Information Network] database^23^), constituted of a different set of patients, practices, and reference population. This makes prevalence rates calculated from both databases incomparable.

The prevalence rate is calculated for each year by dividing the number of newly identified or existing pSS patients by the number of patients of the population in that year.

- $\text{Prevalence rate}=\frac{N\left(\text{patients with}\;\mathrm{new}\;\text{or existing}\;\mathrm{pSS}\;\text{diagnosis}\right)}{N\left(\text{patient years of the population}\right)}\times 1000$

## 3 RESULTS

### 3.1 Patient selection algorithm

Several patient selection approaches (eg, based on diagnoses, comorbidities, diagnostic test results, prescriptions, and disease episode titles) were compared to find the most applicable rules for the phenotyping algorithm. Details of the data set and the final phenotyping algorithm are provided in Supporting Information Appendix.

#### 3.1.1 ICPC codes

Table 2 lists the ICPC codes (including counts) used to include and exclude patients with diseases related to Sjögren's syndrome based on the ACR/EULAR criteria.

**TABLE 2:** Inclusion and exclusion criteria applied to Nivel PCD

Diseases (ICPC code)		N (patients)
*Inclusions*		
Patient has one or more of:	Other musculoskeletal diseases (L99)	347 082
	Other disease eye (F99)	267 361
	Non‐Hodgkin's disease (B72.02)	3674
*Exclusions*		
Patient has one or more of:	Hepatitis (incl. Hepatitis C infection) (D72)	8580
	Other infections of the lungs (R83)	62 091
	HIV (B90)	3045
	Sarcoidosis (B99)	4913
	Graft‐vs‐Host disease (A87)	22 345
	Amyloidosis (T99)	23 526
	IgG4‐related disease (B99)	4913
*Exclusions secondary Sjögren* ^a^		
Patient has:	Rheumatoid arthritis (L88)	31 472

Abbreviation: ICPC, International Classification of Primary Care.

^a^ Two of the three exclusion criteria for secondary Sjögren defined by Pasoto et al,^16^ Systemic Lupus and Systemic Sclerosis, could not be excluded because these are recorded under the ICPC code L99 (“Musculoskeletal disease other”), which is also the ICPC code for Sicca/Sjögren.

#### 3.1.2 Diagnostic test results

Of the diagnostic tests used for diagnosing pSS defined by Shiboski et al,^6^ the NHG lab codes include only the autoantibodies anti‐Ro/SSA. Schirmer's test, salivary flow and ocular staining tests are generally conducted by the Rheumatologist or Ophthalmologist and as such are not recorded in primary care. Therefore test results were not used as input for the patient selection algorithm. After finalizing the patient selection, we did check for how many patients autoantibodies anti‐Ro/SSA values were recorded in Nivel PCD; this was only for four of the 1462 selected pSS patients.

#### 3.1.3 Prescriptions

In addition to the ICPC codes, we assessed the use of Artificial tears, Hydroxychlorochine, Cortisone, Pilocarpine, and Ciclosporin, and the combined use of Artificial tears, Hydroxychlorochine, and Pilocarpine in specific. However, these medications are barely prescribed by the GP in the Netherlands. For example, for all Dutch patients in Nivel PCD in the period 2006 to 2017 (N = 3 056 928), prescription rates for Cortisone (N = 308), Pilocarpine (N = 200), and Ciclosporin (N = 143) are low. When applying the combination of the three prescribed medications to the final patient selection, only 24 of the patients that met the defined pSS selection criteria from Table 2 remained. The (combined) prescription use thus does not seem to be a feasible selection criterion for pSS.

To gain insight in the prescriptions that were used a lot by possible pSS patients, Table 3 shows the prescriptions with the highest recording rates over the complete period. In total 928 different medications were prescribed to the possible pSS patients found in Nivel PCD. Of these, especially artificial tears, proton pump inhibitors (Omeprazole and Pantoprazole), beta blocking agents (Metoprolol), and thyroid hormones (Levothyroxine) were highly used. Apart from artificial tears, these are among the highest used drugs in the general population and are probably related to other morbidities than pSS.

**TABLE 3:** Top 10 of in total 928 unique prescriptions used by pSS patients

ATC code	Description	N (records)
S01XA20	Artificial tears and other indifferent preparations	11 838
A02BC01	Omeprazole	7584
A02BC02	Pantoprazole	5483
C07AB02	Metoprolol	4415
H03AA01	Levothyroxine	4327
B01AC06	Acetylsalicylic acid	4262
C10AA01	Simvastatin	4090
P01BA02	Hydroxychloroquine	3752
C03AA03	Hydrochlorothiazide	2946
N05CD07	Temazepam	2905

Abbreviation: ATC, Anatomical Therapeutic Chemical Classification system for medicines.

#### 3.1.4 Disease episode titles

In total, one of the defined variations of the word “Sjögren” occurred in the disease episode titles of 3259 unique patients. The majority of GPs used the term “Sjogren” (N = 2944), followed by “Sjögren” (N = 256), and various misspellings “Sjorgen” (N = 31), “Sjoegren” (N = 16), “Sogren” (N = 7), “Sjogern” (N = 3), and “Sjorgren” (N = 2).

The distinction between primary and secondary Sjögren was not often explicitly made in the episode texts. For only 71 patients Sjögren was specifically defined as primary (indicated by “prim,” “prim.,” or “primary”) and for 65 patients as secondary (indicated by “sec,” “sec.”, or “secondary”). When the GP was unsure of a patient having Sjögren this was often indicated by a question mark: for example, “Sjögren?” (N = 348). However, as the episode titles are free text fields, the variation in used text strings was high and each patient was assigned to one of the categories manually by taking into account the complete textual context.

Text strings interpreted as primary Sjögren were mainly clear and short statements such as “m. Sjögren,” “morbus Sjögren,” and “Sjögren's syndrome,” without the mention of any secondary diseases (Table 2). Text strings interpreted as perhaps Sjögren contained words such as “suspicion of Sjögren” or “possibly Sjögren,” or the use of a question mark. Text strings interpreted as not or secondary Sjögren clearly stated “no(t) Sjögren,” “secondary Sjögren”, “Sjögren” combined with one of the secondary diseases, or regarded a family member having Sjögren or the patient only being afraid of having Sjögren. This resulted in the following counts per category: (a) “primary Sjögren” (N = 2319); (b) “perhaps primary Sjögren” (N = 672); or (c) “not Sjögren” or explicitly “secondary Sjögren” (N = 268).

#### 3.1.5 Final algorithm

The selection criteria based on the formal ACR/EULAR classification criteria (listed in Table 2), combined with the mention of “Sjögren” (or variations) in the disease episode titles were found to be the most suitable identifiers for pSS in primary care EHRs. To be defined as pSS patient, one or more of the ICPC inclusion criteria should be recorded in the patient journal in the defined period and “Sjögren” (or variations) should be mentioned in the disease episode titles. Only a record of one or more of the inclusion criteria and no mention of “Sjögren” (N = 623 700), or vice versa (N = 729), was not sufficient to be included as a pSS patient. Any patients for which any of the exclusion criteria were recorded were subsequently excluded from the selection. This resulted in a total sample of 1462 plausible pSS patients that were retrieved from Nivel PCD, leading to a prevalence of 0.81 per 1000 patients in 2017.

**FIGURE 1:**
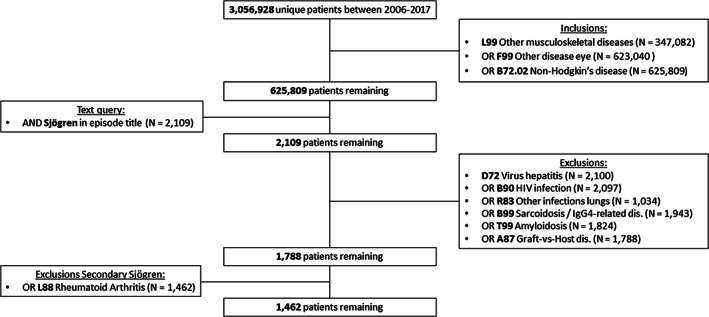
Flowchart inclusion and exclusion criteria applied to Nivel PCD data (cumulative numbers)

The flowchart in Figure 1 shows the inclusion and exclusion rules applied to the total number of patients extracted from the primary care database for the years 2006 to 2017 (N = 3 056 928). Of these, 625 809 patients visited the GP for one or more of the diseases related to Sjögren (L99, F99, or B72.02). Since it is possible for patients to visit the GP for either one or multiple defined diseases in the given period, the flowchart displays cumulative numbers per inclusion and exclusion step instead of absolute numbers per disease (which can be found in Table 2).

First, 347 082 patients were included because they visited the GP for complaints recorded under “Other musculoskeletal diseases” (ICPC code L99). Second, 275 958 additional patients recorded under “Other disease eye” (ICPC code F99) were included, leading to 623 040 patients with codes L99 or F99. Third, an additional 2769 non‐Hodgkin's disease (ICPC code B72.02) patients were included, leading to a total of 625 809 included patients who met at least one of the inclusion criteria.

Of these, only 2109 also had “Sjögren” or any of the defined textual variations mentioned in the disease episode titles, leading to 2109 remaining patients. Of these in total 321 patients were excluded because they visited the GP for one or more of the defined exclusion diseases (D72, B90, R83, B99, T99 or A87), leaving 1788 patients. Finally, 326 of these patients were excluded as these were recorded as having Rheumatoid Arthritis (ICPC code L88), which was defined as a criterion for secondary Sjögren's disease, leaving 1462 pSS patients.

### 3.2 Validating the algorithm

The claims data indicate that on average around 54 000 unique patients per year visit the hospital for a treatment recorded under one of the Sjögren related DRGs or the ICD‐10 code for Sjögren. Based on the estimated global prevalence of 61 per 100 000^7^ and a total Dutch population of 17 million, we would expect slightly over 10 000 patients. Table 4 shows the number of patients for whom Sjögren related DRGs were recorded in the years 2013 to 2017, or who had an ICD‐10 recorded Sjögren diagnosis in the years 2016 to 2017. The majority of the identified patients were treated at the Ophthalmology department, followed by Rheumatology and Internal Medicine.

**TABLE 4:** Number of pSS patients in secondary care

Year	N (unique patients)					Reference population^a^	Population prevalence (per 1000)
	DRG			ICD‐10	Total unique patients (N = 208 545)		
	Rheumatology (N = 10 045)	Internal medicine (N = 1447)	Ophthalmology (N = 201 648)	M35.0, M350 (N = 34 933)			
2013	4740	703	56 862	n.a.	60 995	16 779 575	3.64
2014	5089	629	53 250	n.a.	57 656	16 829 289	3.43
2015	5073	584	50 439	n.a.	54 854	16 900 726	3.25
2016	4870	550	46 044	22 104	50 427	16 979 120	2.97 (1.30)^b^
2017	4896	554	42 277	18 958	46 621	17 081 507	2.73 (1.11)^b^

*Note*: DRG data is available from 2013, 1 year after the implementation of the updated DRG system in 2012.

Abbreviations: DRG, Diagnosis Related Groups, ICD‐10, International Classification of Diseases.

^a^ Retrieved from StatLine Open Data provided by Statistics Netherlands (https://opendata.cbs.nl/statline), retrieved November 2019.

^b^ Prevalence based only on patients with recorded ICD‐10 code for Sjögren.

As the number of Sjögren patients in secondary care defined based on DRG and ICD‐10 codes is higher than expected, we compared the recorded DRGs with the available ICD‐10 codes as the ICD‐10 codes are more explicit diagnoses and DRG codes might be too broad. In the years for which ICD‐10 codes were available (2016 and 2017), much overlap was found between the Sjögren DRGs recorded in the Rheumatology and Internal Medicine departments. For Rheumatology, 4397 of the 4870 (90.3%) patients for which a Sjögren related DRG was recorded also had the ICD‐10 Sjögren diagnosis recorded in 2016. For 2017 this was the case for 4501 of the 4896 patients with a Rheumatology DRG (91.9%). For Internal Medicine 444 of the 550 (80.7%) patients had both the Sjögren related DRG and ICD‐10 diagnosis code in 2016, and 458 of the 554 (82.7%) in 2017. For the Ophthalmology department this overlap was a lot smaller; only 17 806 of the 46 044 (38.7%) patients with a Sjögren DRG in 2016 and 14 596 of the 42 277 (34.5%) patients with a Sjögren DRG in 2017 also had the ICD‐10 code for Sjögren recorded. For the patients for which no Sjögren ICD code was recorded, the ICD code was mainly missing, or referred to an “Unspecified Illness” (R69), Myositis Ossificans Progressiva (M61.19), Congenital malformation syndromes predominantly associated with short stature (Q87.1), or Other disorders of lacrimal gland (H04.1). Especially the latter was highly recorded for patients with a Sjögren DRG at the Ophthalmology department.

To check whether the Sjögren DRGs for each department included any diseases related to Secondary Sjögren, we checked for the presence of ICD10 codes related to the three secondary diseases listed in Table 2 among the patients with Sjögren related DRGs in the period 2016 to 2017. For the Internal Medicine department, none of the patients with a Sjögren DRG was diagnosed with any of the secondary diseases. For the Rheumatology department, 75 unique patients were diagnosed with Rheumatoid Arthritis and ≤10 patients with Systemic Lupus Erythematosus or Systemic Sclerosis. For the Ophthalmology department, ≤10 patients were diagnosed with Systemic Lupus Erythematosus or Systemic Sclerosis and none with Rheumatoid Arthritis.

#### 3.2.1 Linked patients

**FIGURE 2:**
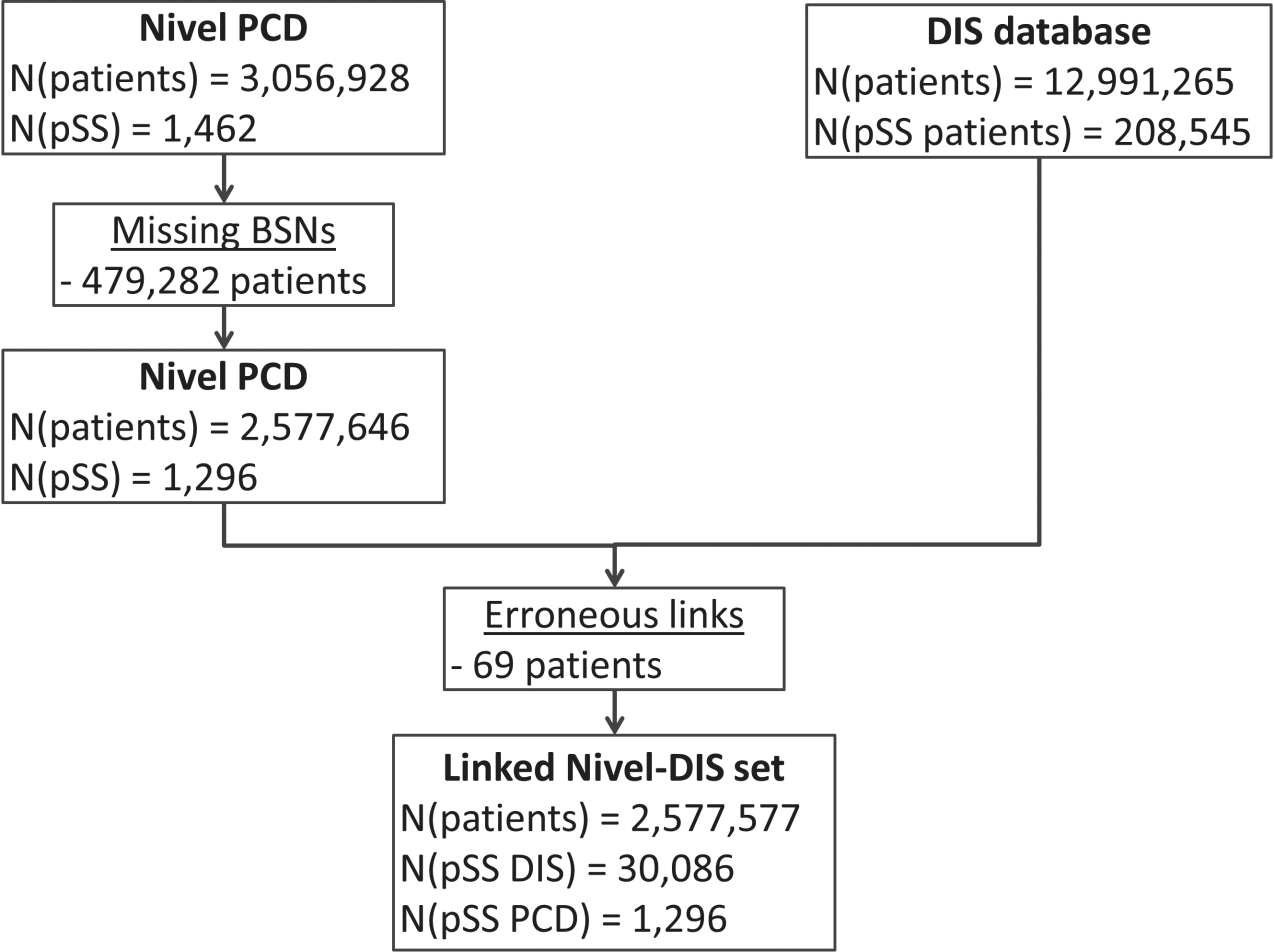
Linkage process

For 208 545 of the 12 991 265 unique patients who visited a medical specialist in any hospital in the Netherlands between 2013 and 2017, a Sjögren related DRG or ICD‐10 code was recorded. In total, 2 577 577 of the 3 056 928 patients included in Nivel PCD could be linked to the secondary care data in the DIS database. Among the linked patients, 30 086 of the initial 208 545 patients with a Sjögren related DRG or ICD‐10 code from the DIS database remained, against 1296 of the 1462 pSS patients found in Nivel PCD, as shown in Figure 2.

#### 3.2.2 Validation scores

The matrix in Table 5 visualizes the performance of the algorithm applied to Nivel PCD by comparing the patients found in Nivel PCD to the formally diagnosed patients in the DIS database. The cells contain the true and false positives and negatives. The number of true positives (Tp) shows that 716 out of the 1296 (55.3%) linked patients that were likely to have pSS in Nivel PCD, indeed visited the hospital medical specialist for a Sjögren related treatment (DRG) between 2013 and 2017 or were recorded as a Sjögren patient (ICD‐10) during a visit to the hospital in 2016 and 2017. A total of 580 of the 1296 linked patients remained unconfirmed based on the DRG dataset, meaning that these pSS patients did not visit the hospital for a Sjögren related treatment in the years 2013 to 2017 or did not receive a formal diagnosis recorded by a specialist (ICD‐10 codes) in the years 2016 to 2017.

**TABLE 5:** Confusion matrix

		Formal pSS diagnosis (DIS)		
		pSS	Non‐pSS	Total
Possibly pSS (Nivel PCD)	pSS	716 *[Tp]*	580 *[Fp]*	1296
	Non‐pSS	29 370 *[Fn]*	2 546 911 *[Tn]*	2 576 281
	Total	30 086	2 547 491	2 577 577

Abbreviations: DIS, Dutch National Insurance Claims Database; Fn, false negatives; Fp, false positives; Nivel PCD, Nivel Primary Care Database; pSS, primary Sjögren's syndrome; Tn, true negatives; Tp, true positives.

Table 5 shows 580 of the 1296 linked patients who were identified as possible pSS patients based on Nivel PCD data were not confirmed based on information from the DIS database. These may not be pSS patients, or pSS patients that did not visit a hospital for a Sjögren related treatment in the years 2013 to 2017. Of the 580 unconfirmed pSS patients in primary care, 213 visited the hospital in the defined period for other DRGs (eg, Cataract (N = 71), Chest pain (N = 65), Perceptive hearing loss (N = 46), or Osteoarthritis of the knee (N = 41)), whereas 367 did not visit the hospital at all. A total of 29370 of the 30 086 patients who visited the hospital for a Sjögren related treatment were not identified as a possible pSS patient in Nivel PCD. The values from the matrix lead to the following performance scores; Accuracy (92.4%), Sensitivity/recall (2.38%), Specificity (99.98%), PPV/precision (55.25%), NPV (98.84%), F_1_‐score (4.56%).

#### 3.2.3 Patient characteristics

Table 6 shows the mean age and gender of the total population included in Nivel PCD and for the selected pSS patients over the years. It also shows the number of new and known pSS patients for each year. The number of new patients in a given year is the number of patients for which “Sjögren” was mentioned for the first time in the ICPC episode title in that year. The total number of patients in a given year is the number of new patients in that year added to the number of patients known from previous years.

**TABLE 6:** Nivel PCD sample and population characteristics

Year	2006	2007	2008	2009	2010	2011	2012	2013	2014	2015	2016	2017	Total
*Total subjects in database*													
Population	164 678	221 887	217 025	288 795	885 226	1 313 607	1 558 192	1 803 696	1 870 279	1 926 817	1 890 690	1 810 851	3 056 928
*Gender*													
Male	81 918	109 253	106 617	142 134	435 822	647 149	767 072	888 692	922 460	952 151	934 120	894 863	
Female	82 755	112 628	110 407	146 660	449 404	666 458	791 120	915 003	947 407	974 600	956 203	915 745	
Unknown	5	6	1	1	0	0	0	1	412	66	367	243	
*Age*													
Mean	37.18	37.80	38.76	39.00	39.07	39.72	39.53	40.05	43.79	44.77	42.86	43.47	
SD	21.94	22.31	22.38	22.41	22.72	22.91	22.87	23.31	82.03	89.80	62.15	68.24	
Selected pSS patients													
New	17	29	39	37	40	61	48	58	56	61	77	68	
Total	888	917	856	993	1033	1094	1142	1200	1256	1317	1394	1462	1462
Non‐patients	163 790	220 970	216 169	287 802	884 193	1 312 513	1 557 050	1 802 496	1 869 023	1 925 500	1 889 296	1 809 389	
Prevalence (per 1000)	‐	‐	‐	‐	‐	0.83	0.73	0.67	0.67	0.68	0.74	0.81	
*Gender*													
Males	100	107	113	118	122	124	133	142	148	157	167	177	177
Females	788	810	843	875	911	970	1009	1058	1108	1160	1227	1285	1285
*Age* ^a^													
Mean	54.74	55.81	56.73	57.62	58.82	59.55	60.35	61.15	62.05	62.81	63.65	64.43	
SD	33.56	33.10	32.49	32.05	31.54	30.89	30.41	29.85	29.37	28.85	28.21	27.78	

Abbreviation: pSS, Primary Sjögren syndrome.

^a^ This is the mean age of the patients included in the database in the concerning year. We do not report mean age in year of first diagnosis here, because the first diagnosis year is unknown for a large group of patients who were diagnosed before inclusion in Nivel PCD (N = 810).

**FIGURE 3:**
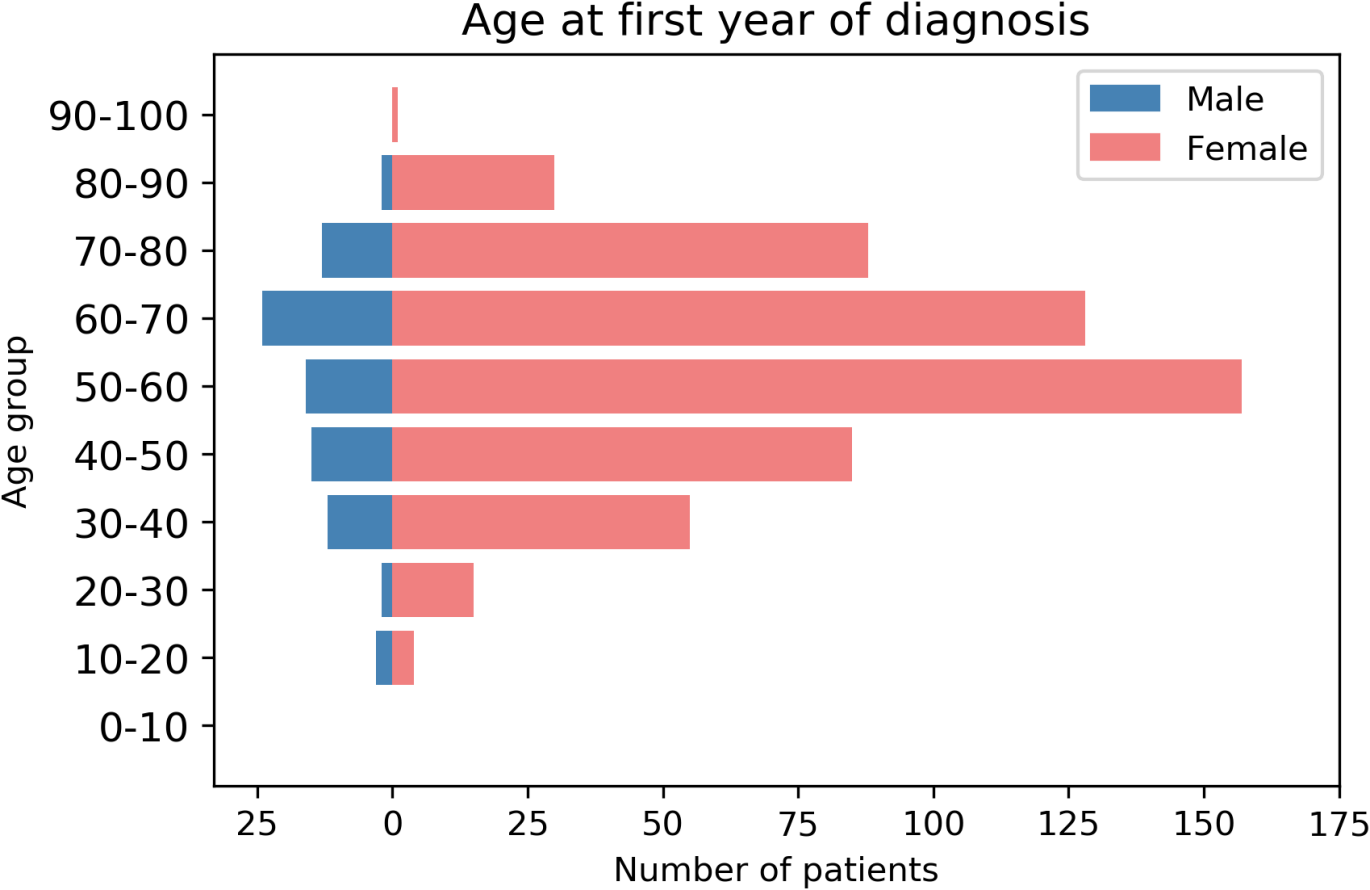
Age distribution at first diagnosis year

As the first year of diagnosis we used the first date in which a record was found in the journal in which “Sjögren” was mentioned in the ICPC text episode title. This date was unknown for 810 pSS patients, probably because the diagnosis was made before the patient had been listed as patient in the practice for which data are included in the database. For the patients for which this date could be retrieved, the majority was between 50 and 70 years of age at the first year of diagnosis (see Figure 3), with a mean of 65.8 years (SD = 15.1).

#### 3.2.4 Prevalence rates

Table 6 displays the prevalence rates, in which the total and new number of pSS patients are compared to the total patient population in Nivel PCD. These rates show the prevalence has slightly increased in the most recent years, after a slight decrease in the first years of the new database. On average the prevalence of pSS patients in Nivel PCD was 0.7‰.

## 4 DISCUSSION

This study illustrates the potential use of routinely recorded primary and secondary care EHR data to identify and validate patients with complex diseases such as pSS. A patient selection algorithm was developed based on known inclusion and exclusion criteria used in the diagnosis of patients with Sjögren's syndrome. ICPC coded diseases combined with keywords extracted from episode text titles were found to be the most suitable for identifying possible pSS patients in primary care, resulting in 1462 possible pSS patients identified in primary care. The patients selected by the algorithm were compared to patients treated for Sjögren's syndrome in secondary care, resulting in a confirmation of 716 of the 1296 linked pSS patients (55.3%).

The first part of our study focused on the question how formal inclusion and exclusion criteria for pSS used by medical specialists in secondary care could be applied to EHR data recorded in primary care. The exact ACR/EULAR classification criteria for pSS could not be easily applied to the available primary care data. The ICPC codes are less granular than the specified ICD‐10 codes used in secondary care, and cover more diseases than the ones specified as a single inclusion or exclusion criterion. In addition, GPs often record only the main ICPC disease codes and not always the more specific sub codes. This complicated the inclusion and exclusion of explicit sub diseases such as Hepatitis C infection, which was now excluded using the overarching main category “Hepatitis.” Another consequence of the broader ICPC codes was that secondary Sjögren's diseases Systemic Lupus Erythematosus and Systemic Sclerosis could not be excluded, as these are both recorded under ICPC code L99 (Musculoskeletal disease other), which is also the code generally used for Sjögren's Syndrome. The translation of the formal disease based classification criteria to criteria applicable to primary care data thus may have resulted in a less precise selection of pSS patients.

Consequently, the selection of pSS patients based on ICPC codes only was not specific enough. Combining the ICPC codes with a mention of Sjögren in the disease episode title narrowed the selection down to more a plausible number of patients. Besides the ICPC disease codes and the episode titles, we examined ATC‐coded medication prescribed by the GP that was expected to be frequently used by pSS patients, and NHG‐coded diagnostic test results conducted by the GP. However, prescription rates for the defined medications were quite low. This could be because this type of medication is not used a lot in the Netherlands, possibly because some are not covered by the general health insurance, or because these are prescribed by specialists in the hospital and not by the GP (and therefore cannot be retraced in our database). With regard to the diagnostic tests, it was found that only one of five tests that can be used to diagnose pSS^6^ is used by GPs in the Netherlands, and recordings of their use are very limited. Although primary care EHRs are quite extensive, only a limited amount of the information needed to apply the formal diagnostic criteria for pSS was available in primary care. Based on the information that was available in the GP records, an alternative phenotyping algorithm could be developed to define a plausible set of pSS patients.

The second part of our study focused on the question of whether DRG and ICD codes retrieved from hospital claims data could be used to validate the primary care algorithm and resulting patient selection. The number of Sjögren related DRGs in the DIS database seems highly inflated when compared to known global prevalence estimates. When using both the DRGs and ICD‐10 codes recorded at the Rheumatology, Internal Medicine, and Ophthalmology departments, the relative number of pSS patients found and the corresponding prevalence rates are much higher than those found in Nivel PCD and reported by Qin et al.^7^

There may be several reasons for this overestimation in secondary care. First, it may be the consequence of strategic recording behavior. DRGs that are used as a basis for reimbursement (as is the case for the DIS database) have been found to be at risk for upcoding.^12^, ^24^ A second reason could be that the recorded DRGs may only be indicative of a suspected Sjögren diagnosis, for which the treatment results turn out to be negative. However, the comparison of DRGs with the ICD10 diagnosis codes recorded at each department for the years 2016 and 2017 showed a high overlap between the DRGs and ICD codes recorded at the Rheumatology and Internal Medicine departments. This may indicate that the DRGs of these departments do not suffer from upcoding and reflect the formal diagnoses recorded by means of the ICD code. This does not seem to apply to the Ophthalmology department, for which less than half of the recorded DRGs overlapped with the ICD codes.

Another reason for the high number of pSS patients recorded in the claims data set might be that the DRGs include cases of primary as well as secondary Sjögren. A check for the presence of ICD10 diagnosis codes related to secondary Sjögren diseases showed that the DRGs recorded at the Rheumatology department included a small number of patients (N = 75) diagnosed with the secondary Sjögren disease Rheumatoid Arthritis. The other secondary diseases were only recorded for very little (N ≤ 10) patients at the Rheumatology and Ophthalmology departments. No patients with secondary Sjögren diseases were included in the DRGs recorded at the Internal Medicine department. This shows that the DRGs include mainly primary and only very few secondary Sjögren's patients. Although DRGs from hospital claims data may not provide sufficiently accurate diagnostic information to be reliably used for the validation of patient selection algorithms, our analyses did show that, especially for Rheumatology and Internal Medicine, DRGs are a suitable alternative for ICD codes when ICD codes are not available.

Despite the high number of recorded Sjögren DRGs, the comparison of pSS patients found in primary care with those treated in secondary care resulted in a relatively low number of confirmed patients. There may be several explanations for this:

1. Some patients found via the algorithm in general practice may not have been referred to specialized care (yet). This is a plausible explanation, as the average time to diagnosis of Sjögren's syndrome, the time it takes for a patient to be referred to a specialist to get a formal diagnosis, is known to be long.
2. Some patients' last visit to the hospital for a Sjögren related treatment had taken place before 2013.
3. Some patients have received secondary care treatment (DRGs) or diagnosis (ICD‐10 codes) other than the ones defined by us.
4. Despite meeting the criteria from the algorithm, some of these patients may not have been pSS patients, meaning the algorithm incorrectly identified some patients as possible Sjögren patients. In order to examine this further, the characteristics of the confirmed and unconfirmed patient groups will be compared (check for significant differences).
5. In spite of claims regulations, DRG groups in claims data may not represent true pSS patients.

In future research we will first focus on exploring and confirming these possible explanations by comparing the primary and secondary care characteristics of the confirmed and unconfirmed pSS patients. Second, we aim to fine‐tune the patient selection algorithm for primary care and the resulting patient selection by studying the characteristics of the pSS patients that were included in the DIS database but that were not found in Nivel PCD based on the initial selection criteria. This may result in additional pSS identifiers in primary care, to be implemented in an improved, more precise algorithm for the selection of pSS patients in general practice. Third, we will develop a timeline displaying the average combined primary and secondary care trajectory of pSS patients in the Netherlands, using the linked Nivel PCD and DIS data of the confirmed pSS patients. This timeline will provide more insight into the used healthcare and the diagnostic process.

When looking at the prevalence rates based on the Dutch primary care database, we see the average prevalence based on our final algorithm (0.7‰) is comparable to the global population prevalence of 0.61‰ reported by Qin et al.^7^ Our mean age at diagnosis (Figure 3) is comparable to the average age of 56.16 years reported by Qin et al.^7^ The female:male ratio in our sample is 7:1, which is to be expected as pSS primarily affects peri‐ and postmenopausal women. Our female:male ratio is lower than the ratio in the prevalence data reported by Qin et al,^7^ which was 11:1. The proportion and characteristics of the pSS patients in primary care identified by the phenotyping algorithm are thus mostly in line with those reported in literature. The number of pSS patients in secondary care, however, highly exceeded the number expected based on the general population prevalence. Even when using only ICD‐10 codes, which might be a more accurate source of diagnostic information, the prevalence found for the Netherlands still exceeds global estimates. There is not enough information to assess whether this discrepancy can be attributed to the sources and methods used to identify pSS patients in secondary care or the possibility that literature reported global prevalence rates might not be accurate for the Netherlands. This has a major impact on our study results in that it is unclear whether insurance claims records are a suitable source to compare and confirm the results obtained from primary care data with and, consequently, we cannot draw unambiguous conclusions regarding the quality of our patient selection and the developed phenotyping algorithm.

This study shows the possibilities of using EHR data for studying complex medical conditions. It is clear that population‐based health records provide a lot of longitudinal medical information and insight in the use of care for a large range of diseases. However, the study of patients with low prevalence, uncoded diseases is more challenging, as those cannot be as easily identified from primary care data as patients with more general diseases. The lack of a granular coding system for symptoms and diseases also makes it difficult to apply diagnostic criteria used in secondary care to data recorded in primary care. The possibility to link primary to secondary care databases on patient level allows one to (iteratively) try different patient selection algorithms and compare those to patients referred to specialized care, and to study patient and care characteristics in primary care of patients thus far only known in secondary care. As such, these combined medical data should be considered a rich source of information for the epidemiological study of low prevalence, complex diseases, patients' early symptoms, diagnosis paths, and overall treatment trajectories in primary and eventually secondary care. However, without the formal diagnostic information required to validate the developed phenotyping algorithm and patient selection, we have insufficient information to affirm that routine EHR data is fit for the identification and study of patients with complex diseases such as pSS.

## CONFLICT OF INTEREST

The authors do not have any conflicts of interest to declare.

## Supporting information


**Appendix S1**: SUPPORTING INFORMATION
