# Supplementary material for: Fitness for purpose of routinely recorded health data to identify patients with complex diseases: The case of Sjögren's syndrome
Source: Learn Health Syst. 2020 Sep 8;4(4):e10242. doi: 10.1002/lrh2.10242 (PMC7556429; doi:10.1002/lrh2.10242)
Supplement: Supplementary file 1 — Appendix S1: SUPPORTING INFORMATION [file LRH2-4-e10242-s001.docx]

# Appendix A. Data set and phenotyping algorithm details

## Dataset description

The dataset covers GP recorded medical information on patient level for **3,056,928** patients enlisted in any practice included in Nivel PCD at any moment during the period **2006-2017**.

The data set includes data on the **topics**: patient, practice, journals, prescriptions, episodes, and test results. Data for each topic can be analyzed on patient level using pseudonymized **patient IDs**. Practice information can be analyzed based on the pseudonymized **practice ID**.

| **Topic** | **Description** | **Variables** |
| --- | --- | --- |
| Patient | All patients enlisted in any Nivel PCD practice at any moment in defined period. | - Patient ID - Practice ID - Year of birth - Gender - Date in practice from - Date in practice until |
| Practice | All practices included in Nivel PCD at any moment in defined period. | - Practice ID - Practice type: unknown, solo, duo, health center - Practice size |
| Journals | All ICPC coded GP contacts per patient. | - Patient ID - Practice ID - ICPC: disease diagnosis code related to contact - ICPCepi: disease diagnosis code for episode under which contact was recorded - Date: recording date journal entry |
| Prescriptions | All ATC coded prescriptions of enlisted patients. | - Patient ID - Practice ID - ATC: prescription code - Prescriber: employee type - First prescriber: care provider that made the first prescription - Frequency of use - Amount prescribed - Repeat prescription: indicator for repeat prescription - Date: prescription date - End date: final date of the prescription |
| Episodes | All ICPC coded disease episodes of enlisted patients. | - Patient ID - Practice ID - Title: disease episode title - ICPC: diagnosis code related to episode - Epistart: start date of episode - Epistop: stop date of episode |
| Results | All NHG coded diagnostic test results conducted at the GP’s office. | - Patient ID - Practice ID - NHG code: diagnostic test result code - Result value: outcome of diagnostic test - Result unit: measurement unit of diagnostic test - Date: testing date |

## Phenotyping algorithm

Patients = all patients enlisted in any Nivel PCD practice at any moment during the years
 2006-2017

Result = possible pSS patients based on the input data

Journal_To_Include = (L99 **or** F99 **or** B72.02)
Episode_To_Include = ("sjogren" **or** "sjorgen" **or** "sjogern" **or** "sjögren" **or** "sjorgren" **or**
 "sjoegren" **or** "sogren")
Journal_To_Exclude = (D72 **or** B90 **or** R83 **or** B99 **or** T99 **or** A87 **or** L88)

**for** patient **in** Patients:
 **if** (patient.Journal **in** Journal_To_Include **and** patient.Episode **contains** Episode_To_Include)
 **and not** (patient.Journal **in** Journal_To_Exclude):
 **add to** Result
